# RETRACTED: Fahmy et al. Intranasal Niosomal In Situ Gel as a Promising Approach for Enhancing Flibanserin Bioavailability and Brain Delivery: In Vitro Optimization and Ex Vivo/In Vivo Evaluation. *Pharmaceutics* 2020, *12*, 485

**DOI:** 10.3390/pharmaceutics16020189

**Published:** 2024-01-29

**Authors:** Usama A. Fahmy, Shaimaa M. Badr-Eldin, Osama A. A. Ahmed, Hibah M. Aldawsari, Singkome Tima, Hani Z. Asfour, Mohammed W. Al-Rabia, Aya A. Negm, Muhammad H. Sultan, Osama A. A. Madkhali, Nabil A. Alhakamy

**Affiliations:** 1 Department of Pharmaceutics, Faculty of Pharmacy, King Abdulaziz University, Jeddah 21589, Saudi Arabia; uahmedkauedu.sa@kau.edu.sa (U.A.F.); oaahmed@kau.edu.sa (O.A.A.A.); haldosari@kau.edu.sa (H.M.A.); nalhakamy@kau.edu.sa (N.A.A.); 2 Advanced Drug Delivery Research Group, Faculty of Pharmacy, King Abdulaziz University, Jeddah 21589, Saudi Arabia; 3 Department of Pharmaceutics and Industrial Pharmacy, Faculty of Pharmacy, Cairo University, Cairo 11562, Egypt; 4 Center of Excellence for Drug Research and Pharmaceutical Industries, King Abdulaziz University, Jeddah 21589, Saudi Arabia; 5 Department of Medical Technology, Faculty of Associated Medical Sciences, Chiang Mai University, Chiang Mai 50200, Thailand; singkome.tima@cmu.ac.th; 6 Department of Medical Microbiology and Parasitology, Faculty of Medicine, King Abdulaziz University, Jeddah 21589, Saudi Arabia; hasfour@kau.edu.sa (H.Z.A.); mwalrabia@kau.edu.sa (M.W.A.-R.); 7 Department of Pharmacognosy, Faculty of Pharmacy, Zagazig University, Zagazig 44518, Egypt; negma@uwindsor.ca; 8 Department of Pharmaceutics, College of Pharmacy, Jazan University, Jazan 45142, Saudi Arabia; mhsultan@jazanu.edu.sa (M.H.S.); omadkhali@jazanu.edu.sa (O.A.A.M.); 9 King Fahd Medical Research Center, King Abdulaziz University, Jeddah 21589, Saudi Arabia

This journal retracts the article “Intranasal Niosomal In Situ Gel as a Promising Approach for Enhancing Flibanserin Bioavailability and Brain Delivery: In Vitro Optimization and Ex Vivo/In Vivo Evaluation” [1], cited above.

Following publication, concerns were brought to the attention of the publisher regarding the overlap of images.

Adhering to our complaint’s procedure, an investigation was conducted by the Editorial Office and the Editorial Board that confirmed the overlap of an image (Figure 5) with the prior [2] and later publications [3].

While the authors fully cooperated with the Editorial Office during the investigation, they were unable to satisfactorily explain the overlap of figures nor could the required quality standards of raw images in order to consider a correction as per the journals original image requirements policy be met (https://www.mdpi.com/journal/pharmaceutics/instruc-tions#oriimages). As a result, the Editorial Board and EIC were unable to confirm the reliability of the findings and subsequently decided to retract this paper.

This retraction was approved by the Editor-in-Chief of the journal *Pharmaceutics*.

The authors did not agree to this retraction.
